# Financial risk protection of Thailand’s universal health coverage: results from series of national household surveys between 1996 and 2015

**DOI:** 10.1186/s12939-020-01273-6

**Published:** 2020-09-21

**Authors:** Viroj Tangcharoensathien, Kanjana Tisayaticom, Rapeepong Suphanchaimat, Vuthiphan Vongmongkol, Shaheda Viriyathorn, Supon Limwattananon

**Affiliations:** 1 International Health Policy Program, Ministry of Public Health, Nonthaburi, Thailand; 2 Division of Epidemiology, Department of Disease Control, Ministry of Public Health, Nonthaburi, Thailand; 3 Faculty of Pharmaceutical Science, Khon Kaen University, Khon Kaen, Thailand

**Keywords:** Universal Health Coverage, Financial risk protection, catastrophic health spending, Health impoverishment, Thailand

## Abstract

**Background:**

Thailand, an upper-middle income country, has demonstrated exemplary outcomes of Universal Health Coverage (UHC). The country achieved full population coverage and a high level of financial risk protection since 2002, through implementing three public health insurance schemes. UHC has two explicit goals of improved access to health services and financial protection where use of these services does not create financial hardship. Prior studies in Thailand do not provide evidence of long-term UHC financial risk protection. This study assessed financial risk protection as measured by the incidence of catastrophic health spending and impoverishment in Thai households prior to and after UHC in 2002.

**Methods:**

We used data from a 15-year series of annual national household socioeconomic surveys (SES) between 1996 and 2015, which were conducted by the National Statistic Office (NSO). The survey covered about 52,000 nationally representative households in each round. Descriptive statistics were used to assess the incidence of catastrophic payment as measured by the share of out-of-pocket payment (OOP) for health by households exceeding 10 and 25% of household total consumption expenditure, and the incidence of impoverishment as determined by the additional number of non-poor households falling below the national and international poverty lines after making health payments.

**Results:**

Using the 10% threshold, the incidence of catastrophic spending dropped from 6.0% in 1996 to 2% in 2015. This incidence reduced more significantly when the 25% threshold was applied from 1.8 to 0.4% during the same period. The incidence of impoverishment against the national poverty line reduced considerably from 2.2% in 1996 to approximately 0.3% in 2015. When the international poverty line of US$ 3.1 per capita per day was applied, the incidence of impoverishment was 1.4 and 0.4% in 1996 and 2015 respectively; and when US$ 1.9 per day was applied, the incidence was negligibly low.

**Conclusion:**

The significant decline in the incidence of catastrophic health spending and impoverishment was attributed to the deliberate design of Thailand’s UHC, which provides a comprehensive benefits package and zero co-payment at point of services. The well-founded healthcare delivery system and favourable benefits package concertedly support the achievement of UHC goals of access and financial risk protection.

## Background

Universal Health Coverage, as committed to by UN Member States in the Sustainable Development Goals (SDG), can contribute to health equity if it is properly designed and implemented [1]. The two explicit objectives of UHC, achieving equitable access to quality health services and ensuring financial risk protection, are key for the overall goal of good health and well being for all, and for other health targets in the SDGs, such as mortality reduction and prevention of premature mortality from non-communicable diseases.

After four decades of health infrastructure development and three decades of extending financial risk protection targeting different population groups with a comprehensive benefits package, Thailand finally achieved UHC in 2002 [1, 2] when the whole population was covered by one of the three public health insurance schemes: (1) the Civil Servant Medical Benefit Scheme (CSBMS) for government employees and retirees and their dependents; (2) Social Health Insurance (SHI) for private-sector employees; and (3) the Universal Coverage Scheme (UCS) for the remaining 47 million population (75% of the total population) who are not covered by CSMBS and SHI.

The UCS, launched in 2002, is financed by general tax revenue through annual budget allocation. The SHI is financed by tri-partite payroll contributions, equally shared by the employee, employer and government, while the CSMBS is financed by general tax revenues [3]. Key characteristics of these three main schemes are described in Table 1.

**Table 1:** Key characteristics of the three main public health insurance schemes in Thailand as of 2020

Insurance scheme	Population coverage	Source of revenue	Mode of provider payment	Access to service
Civil Servant Medical Benefit Scheme (CSMBS)	9%, government employees plus dependants (parents, spouse, and up to 2 children)	General tax, non-contributory scheme	Fee for service, direct disbursement to mostly public providers and Diagnostic Related Groups (DRG) for inpatient treatment	Free choice of public providers
Social Health Insurance (SHI)	16%, private sector employees, excluding dependants	Tripartite contribution, equally shared by employer, employee and the government	Inclusive capitation for both outpatient and inpatient plus additional adjusted payments for accident and emergency and high-cost care	Registered public and private contractors
Universal Coverage Scheme (UCS)	75%, the rest of the ‘Thai’ population not covered by the SHI and the CSMBS	General tax	Capitation for outpatients and global budget plus DRG for inpatients	Registered contractors, notably the network of public hospitals (Contracting Unit for Primary Care)

Source: Tangcharoensathien et al. [4]

The achievement of UHC in Thailand is remarkable, in terms of health-utilisation outcomes and economic merit [5]. The UCS resulted in a reduction of the probability that an ill person would not receive formal treatment and an increased probability of the use of both outpatient (OP) and inpatient (IP) services at public hospitals; the increases in OP utilisation were greatest amongst the poorest part of the population [6]. A recent study suggested that the incidence of catastrophic spending (using household health spending exceeding 10% of household consumption as a benchmark) in Thailand in 2010 was approximately 3%, around fourfold lower than the global incidence of 12% [7].

Notwithstanding these studies, knowledge gaps remain. For instance, research that details the long-term sustainable success of UHC, not just a snapshot assessment, is still lacking. Also, prior research on UHC mostly investigated financial risk protection through the assessment of catastrophic health spending; while impoverishment was not reported in detail.

This study analysed the 15-year trend, biennially between 1996 and 2006 and annually between 2007 and 2015, which covered pre-UHC in 2002 and post-UHC eras after 2002. We analyzed (i) the incidence of catastrophic health expenditure, as measured by out-of-pocket expenditure on health greater than 10 and 25% of total household consumption expenditure; and (ii) the incidence of impoverishment from health payments by households as assessed by three key poverty lines, namely Thailand national poverty line and two international poverty lines using purchasing power parity (PPP) at US$1.9 and US$3.1 per person per day [8, 9]. Finally, this paper explains some determinants, particularly benefits package designs and health systems factors, which contribute to the improved financial risk protection experienced in Thailand.

## Methods

### Data source

Data were retrieved from a series of annual national representative household socioeconomic surveys, conducted by the National Statistical Office of Thailand, between 1996 and 2015. It should be noted that between 1996 and 2006 the SES was conducted biennially, but since 2007 it became an annual survey. However, in this study, the scope of the analysis covered 1996 to 2015, where the most completed data are available. The SES used structured interviews as the main data collection technique. Approximately 52,000 households were recruited in each survey and were divided into 12 equal portions for 12 monthly surveys covering the whole year. That is, about 4333 households were interviewed in January and another 4333 households were interviewed in February, and so forth throughout the year. Surveys undertaken throughout the year is a good practice, which prevents seasonal variations of household income and expenditure. A stratified two-stage random sampling was conducted.

A number of household attributes were collected including household income, consumption expenditure, amount of capital, changes in assets and debts, ownership of durable goods, housing characteristics and monetary expenditure at point of health services. The time reference is 1 month for questions about illnesses, OP utilisation and self-medication. Whereas, the reference for IP utilisation was 1 year. The World Health Organization (WHO) recommends using a one-month and 12 months recall period for outpatient visit and hospitalization respectively in a household survey [10].

### Data analysis

Descriptive statistics were applied to assess (i) the incidence of catastrophic payment, and (ii) the incidence of household impoverishment from healthcare spending. A wealth index which distinguishes rich and poor households was created by a principal component analysis technique through ownership of durables and housing characteristics [11]. The index divided households into quintiles, where the first quintile represented the poorest households while the fifth quintile represented the richest ones. The annual incidence of catastrophic and impoverishment were stratified by household wealth quintiles, geographical regions (Greater Bangkok, Central, North, Northeast and South), and urban-rural areas.

The following operational definitions were used in the analysis. The out-of-pocket payment was defined as a sum of medical and health-related expenditure at point of service and aggregated at the household level [12]. The scope of OOP in this study encompassed the following components: (i) medical expenditure spent during the previous month for non-admission health services which included expenditure on self-medication, traditional or herbal drugs, contraceptives and condom, vitamins, first-aid kits and other medical equipment; (ii) OP care expenditure including dental care and optometric care in the previous month in all facility types (such as public health centres, public hospitals, private clinics and private hospitals); and (iii) IP care expenditure during the past 12 months in all facility types.

The incidence of catastrophic health payment was calculated from the sum of households making catastrophic payments if the share of healthcare spending in a given household exceeded the thresholds, using 10 and 25% of total household consumption expenditure, divided by the total number of sample households.

Whether the household became impoverished from health payments was assessed by comparing total expenditure before and after health payments against the national poverty line, updated regularly by the Office of the National Economic and Social Development Broad, and two international poverty lines using PPP US$ 1.90 and US$ 3.10. Thai Baht was used when applying the national poverty line, whereas international dollars were used when calculating impoverishment against international poverty lines.

To estimate impoverishment, the following procedures were applied. First, the number of households whose expenditure was below the poverty line prior to health payment was estimated and was defined as ‘H-pre’. Second, healthcare payment was subtracted from the total household expenditure. The number of poor households after subtracting OOP was defined as ‘H-post’. Impoverished households are the number of additional poor households after health payments, and its incidence is equivalent to H-post minuses H-pre. Details of the Thailand poverty line during the study period by geographical regions are presented in Table 2.

**Table 2:** Thailand national poverty line (US$ per capita per month) by geographical regions and areas

Geography		1996	1998	2000	2002	2004	2006	2007	2008	2009	2010	2011	2012	2013	2014	2015
Greater Bangkok	Urban	74.27	51.35	53.88	51.21	57.41	66.31	74.32	80.88	78.05	86.98	95.15	96.33	99.16	96.47	91.45
	Total	74.27	51.35	53.88	51.21	57.41	66.31	74.32	80.88	78.05	86.98	95.15	96.33	99.16	96.47	91.45
Central	Urban	63.83	45.74	48.15	46.23	52.73	61.76	69.40	76.73	74.02	83.19	90.65	91.66	95.53	92.11	87.67
	Rural	55.25	40.53	42.44	40.32	45.84	53.94	60.59	67.93	65.85	74.71	81.23	82.34	85.52	82.51	77.54
	Total	58.09	42.29	44.41	42.49	48.52	57.15	64.31	71.74	69.48	78.57	85.60	86.74	90.32	87.19	82.55
North	Urban	56.99	39.93	42.19	40.30	45.15	52.53	59.13	65.59	63.50	71.60	78.81	79.70	82.97	80.46	75.73
	Rural	43.85	31.27	32.47	31.28	35.69	42.30	48.42	54.71	53.07	60.53	66.32	66.71	70.39	68.74	64.83
	Total	46.53	33.03	34.48	33.35	38.10	45.20	51.61	58.12	56.51	64.37	70.84	71.61	75.32	73.50	69.41
Northeast	Urban	52.78	38.09	39.55	37.81	42.83	50.33	57.20	63.70	62.18	70.95	77.50	77.76	81.51	79.51	75.12
	Rural	40.68	30.23	31.35	29.82	33.51	40.80	47.27	53.94	52.14	60.10	66.46	66.87	70.14	68.68	65.03
	Total	42.57	31.49	32.72	31.32	35.47	43.04	49.74	56.50	54.92	63.27	69.86	70.39	73.98	72.51	68.75
South	Urban	63.81	45.25	47.13	45.48	51.24	59.83	67.65	74.89	74.04	83.24	91.51	92.81	96.44	93.55	88.76
	Rural	49.52	34.69	36.11	34.72	40.16	49.22	55.60	62.93	61.17	69.33	76.57	77.43	80.44	78.64	73.80
	Total	52.63	37.04	38.64	37.36	43.09	52.24	59.17	66.62	65.30	73.99	81.73	82.90	86.27	84.21	79.52
Whole country	Urban	64.63	45.51	47.65	45.47	51.24	59.60	67.04	73.81	71.51	80.34	87.78	88.62	92.00	89.17	84.44
	Rural	45.84	33.36	34.76	33.29	37.94	45.69	52.19	59.13	57.38	65.59	72.08	72.82	76.17	74.26	70.04
	Total	51.52	37.07	38.77	37.37	42.73	51.07	58.13	65.20	63.42	72.11	79.20	80.16	83.70	81.50	77.20

Source: 1. NSO, Thailand [8]

Note: Exchange rate as of January 2020 as defined by the World Bank

## Results

### Profile of the samples

The majority of households resided in rural areas (52–69% of total households). About one-third of the households were in the north-eastern region and one-fourth in central and northern regions whereas only 10–14% were in the southern region and Greater Bangkok, Table 3. These profiles did not significantly change between 1996 and 2015.

**Table 3:** Percentage distribution of sample households by geographical region and areas between 1996 and 2015

Year	Geographical regions					Geographical areas		Total number of sample households
	Greater Bangkok	Central	North	Northeast	South	Urban	Rural	
1996	11.75	22.32	20.47	32.52	12.94	30.83	69.17	15,037,898
1998	11.97	22.47	19.99	32.50	13.07	31.18	68.82	15,758,198
2000	12.19	22.58	19.78	32.36	13.09	31.57	68.43	16,086,398
2002	12.43	22.67	19.50	32.25	13.15	32.56	67.44	16,323,070
2004	12.39	22.94	19.70	31.91	13.06	32.61	67.39	16,765,049
2006	10.76	24.58	19.57	32.07	13.02	31.61	68.39	18,051,358
2007	10.78	24.75	19.40	31.99	13.09	31.78	68.22	18,178,247
2008	10.37	24.48	19.71	32.46	12.99	32.11	67.89	18,993,547
2009	10.31	24.19	19.74	32.48	13.27	33.13	66.87	19,579,220
2010	10.24	24.24	19.71	32.44	13.36	36.00	64.00	19,740,866
2011	9.84	24.57	19.67	32.39	13.53	36.19	63.81	19,985,866
2012	9.77	24.63	19.64	32.39	13.57	36.18	63.82	20,068,020
2013	9.71	24.66	19.60	32.37	13.65	36.14	63.86	20,167,519
2014	12.33	29.84	18.30	26.49	13.03	46.28	53.72	20,601,044
2015	13.66	29.77	17.86	25.92	12.78	47.52	52.48	21,325,999

### Incidence of catastrophic health expenditure

**Fig. 1:**
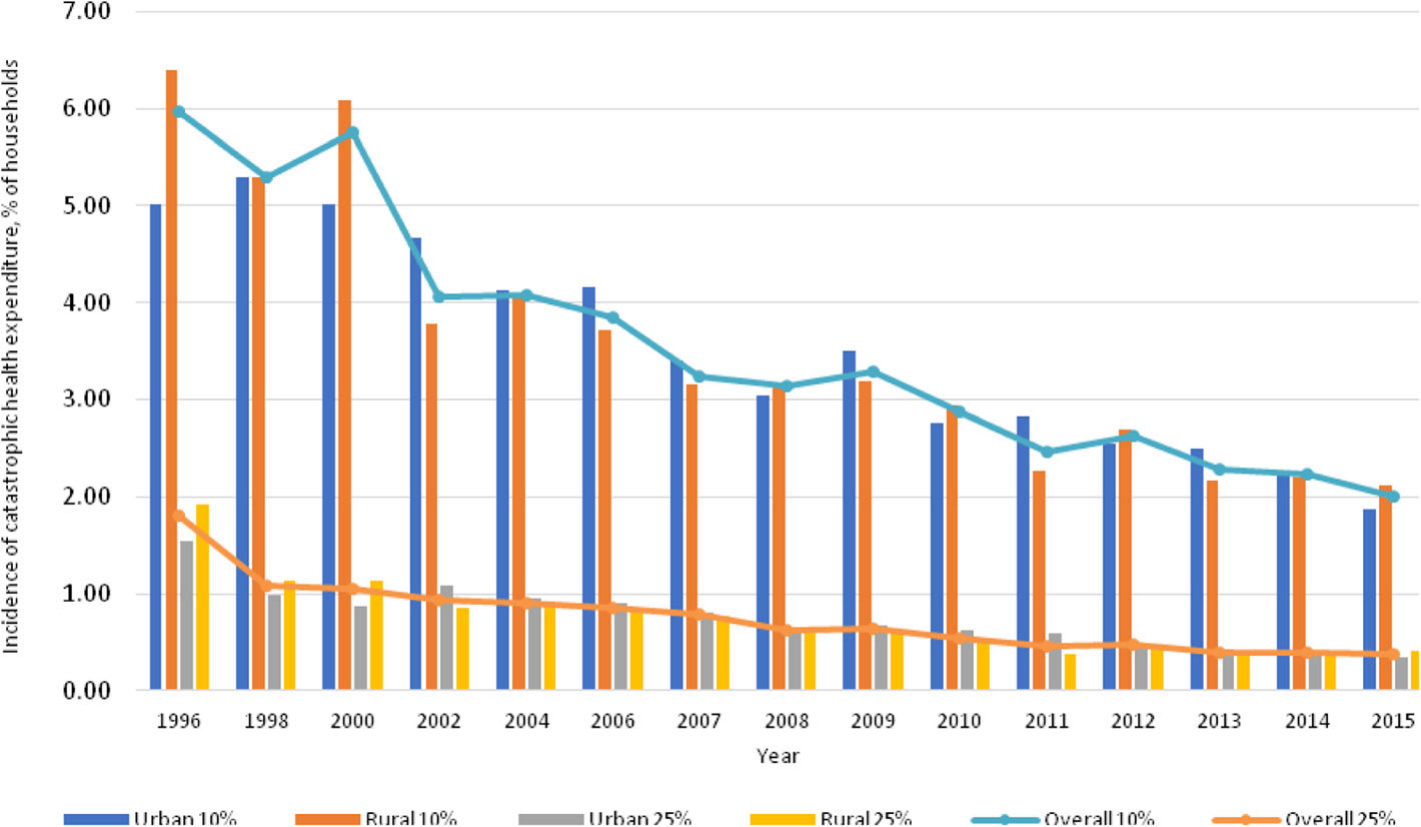
Incidence of catastrophic health expenditure between 1996 and 2015

When the 10% threshold was applied, the incidence of catastrophic health spending slightly decreased during the pre-UHC period from 6% in 1996 to 5.7% in 2000. After the implementation of UHC, catastrophic expenditure dropped dramatically from 4.1% in 2002 to 2.0% in 2015 (about a 50% reduction), see Fig. 1. Using the 25% threshold, the incidence was much lower than when using the 10% threshold. A declining trend was observed from 1.8% in 1996 to 0.4% in 2015. In terms of urban-rural differentials, before the UHC era, the incidence of catastrophic payments among rural households was much higher than in urban households. After UHC was achieved, the urban-rural gap of catastrophic health spending diminished over time. In 2014, we observed the nearly zero urban-rural gaps in household catastrophic health spending.

Households living in the Greater Bangkok area had a higher incidence of catastrophic health spending than other in regions, and the southern region came second. In general, a declining trend was observed in all regions.

**Fig. 2:**
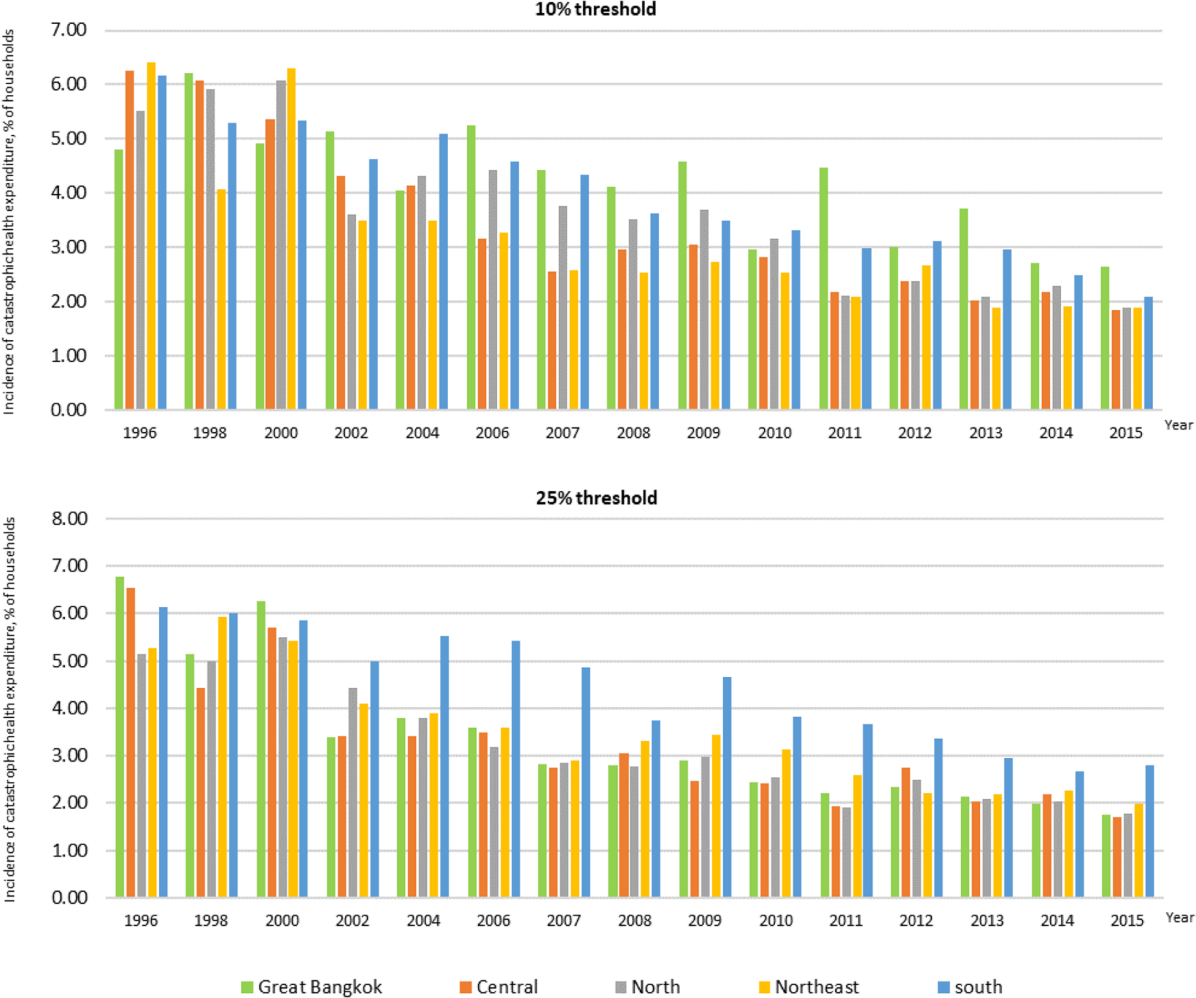
Incidence of catastrophic health expenditure by geographical regions between 1996 and 2015

When the 25% threshold was applied, the incidence of catastrophic health spending was not remarkably different from when the 10%-threshold was applied. The regional difference in the incidence of catastrophic health spending became smaller over time, Fig. 2.

**Fig. 3:**
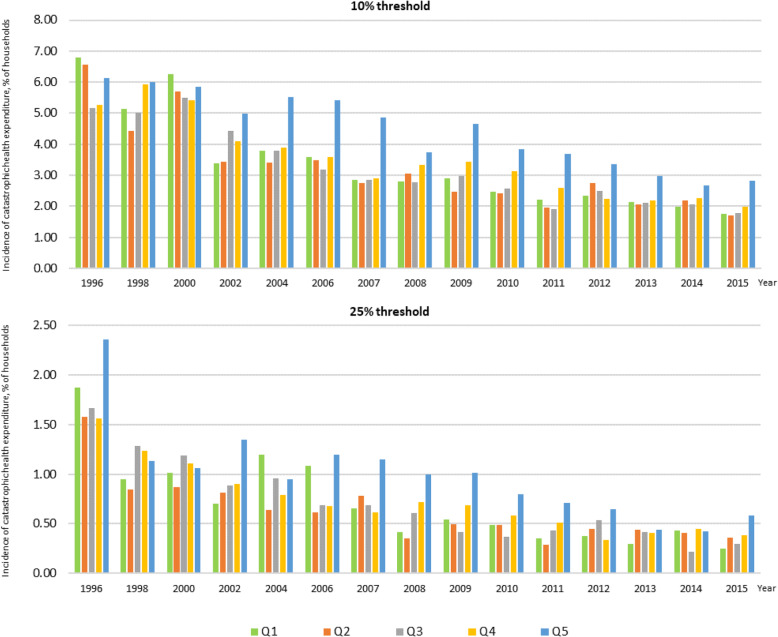
Incidence of catastrophic health expenditure by household asset quintiles between 1996 and 2015

When households were stratified by asset quintiles, the incidence of catastrophic payments dropped drastically after the roll-out of UHC in 2002, in both the richest and poorest quintiles. The richest quintile experienced the greatest incidence of catastrophic spending compared to other quintiles in almost all years observed, Fig. 3.

### Incidence of healthcare impoverishment

The poverty incidence after spending for healthcare, measured by the percentage of households living below the national poverty line, increased from 32.9% in 1996 to about 38.5% in 2000. After UHC was achieved in 2002, poverty incidence decreased by about six-fold to 6.6% in 2015. The incidence of impoverishment as a result of payment for medical bills in 2015 also shrank by four-fold, from 1.3% in 2002 at the beginning of UHC, to approximately 0.3% in 2015, Fig. 4.

**Fig. 4:**
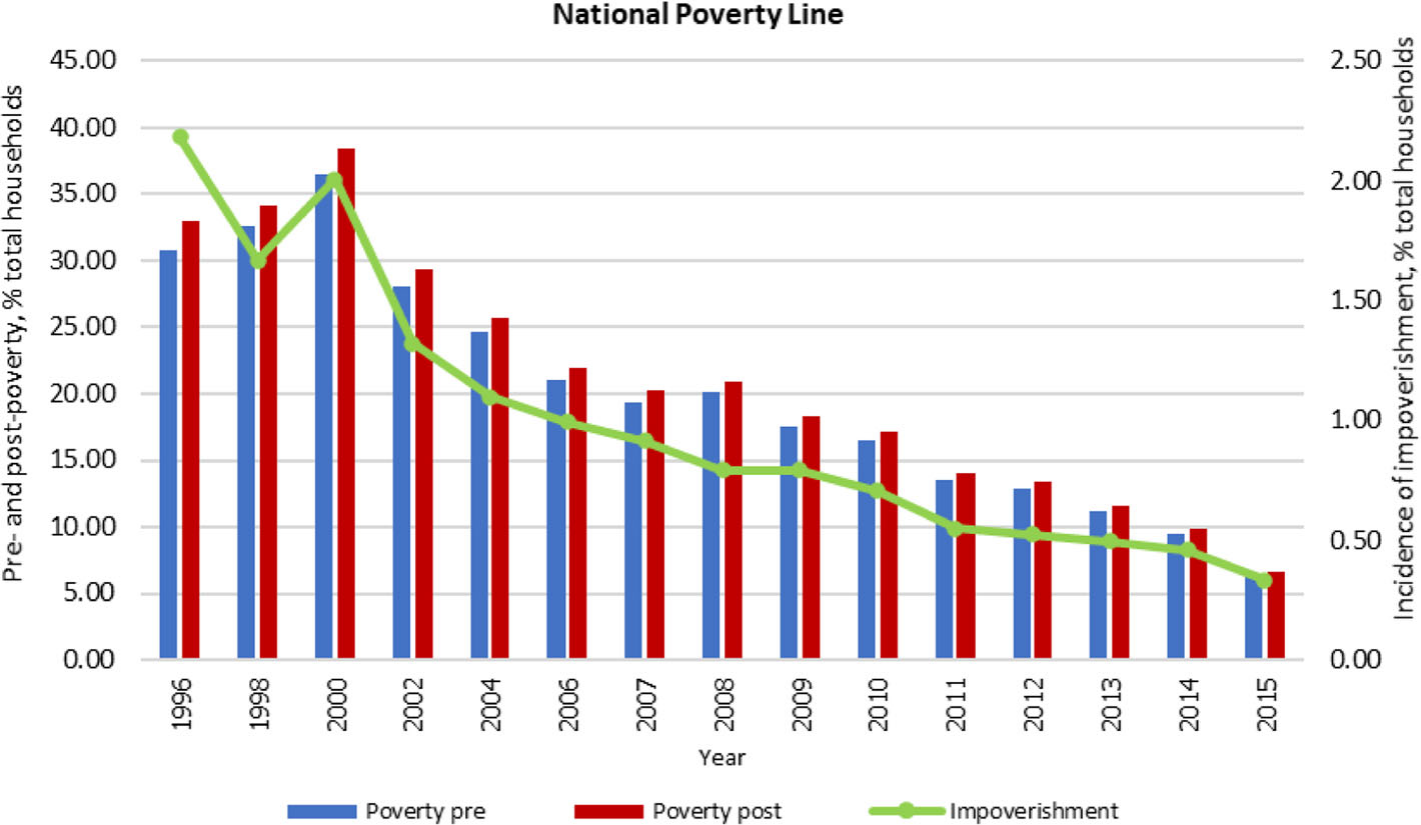
Incidence of households with impoverishment using national poverty line, % total households

**Fig. 5:**
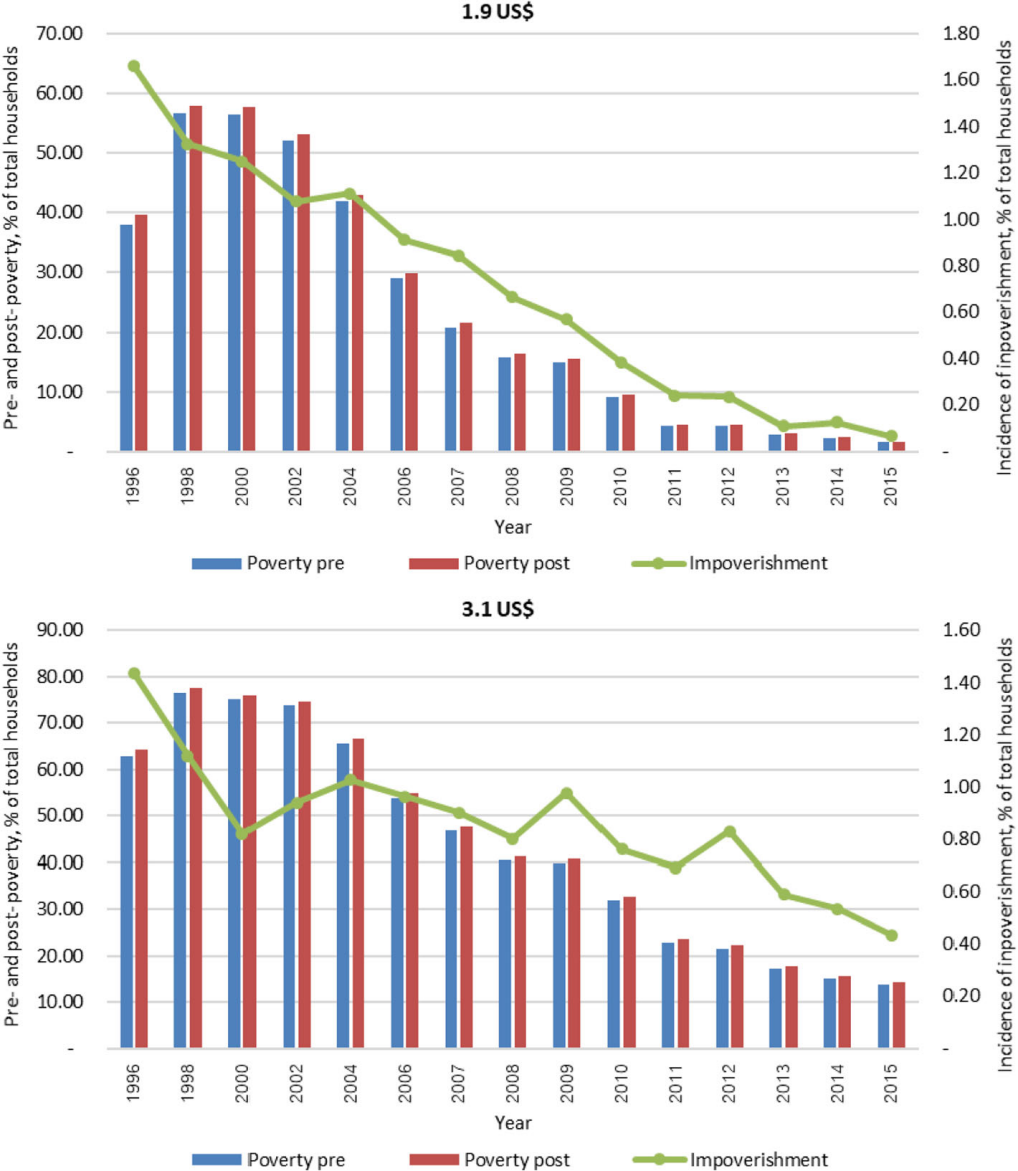
Incidence of impoverishment using international poverty lines (US$ per capita per day), % total households

In 1996 the incidence of impoverishment was about 1.4% (using an international poverty line of US$ 3.1 as a reference) and 1.7% (US$ 1.9 as a reference). After 2002, the trend hugely decreased despite some fluctuations. The incidence of health impoverishment using US$3.1 and US$ 1.9 poverty lines greatly reduced after 2002 when UHC was launched. The incidence of impoverishment appeared to be the smallest in 2015 (0.07% with US$ 1.9 used as the reference), Fig. 5.

## Discussion

UHC as proposed by the World Health Organization is that ‘all’ people and communities are able to access essential health services of sufficient quality, while the government ensures that the use of such services does not expose the users to financial hardship [13]. This study clearly confirms that Thailand’s UHC achieved a high level of financial risk protection against catastrophic health spending and impoverishment from health payments by households and reaffirms the negative correlation between public health insurance coverage and incidence of catastrophic payments [7]. The percentage of households in Thailand encountering catastrophic health spending and healthcare impoverishment was on par with several high-income countries in Europe, North America and Oceania; for instance, Austria, France and Germany [7, 14].

Prior to UHC in 2002, all Thai citizens, including the rural poor, had adequate access to health services at the district level which provided primary and secondary services. As a result of successive governments’ investment in the health delivery system since the 1970s, Thailand had achieved full geographical coverage of a district health system nation-wide by the mid 1990s. The district health system consists of a district hospital of 10–120 beds and a network of 10–15 health centres. A health centre serves a catchment area of five thousand people. District health systems are the backbone for equitable access to services for all populations [4]. In parallel to the health delivery systems expansion, successive governments introduced financial risk protection schemes through targeting different population groups such as low-income households since 1975, the informal sector since 1984 and private sector employees since 1991 [15], until UHC was achieved in 2002. These financial risk protection schemes prior to and after UHC in 2002 provided a comprehensive benefits package [16] with minimum co-payments which reduced household out-of-pocket payment significantly from 34% of Current Health Expenditure (CHE) in 2000 prior to UHC to 11% of CHE in 2017 [17]. Access to care through district health systems, the so called “closed-to-client setting” [18] foster equity in access and benefit incidence [19]. One study also shows equity in maternal and child health services coverage as a result of district health systems [20].

**Fig. 6:**
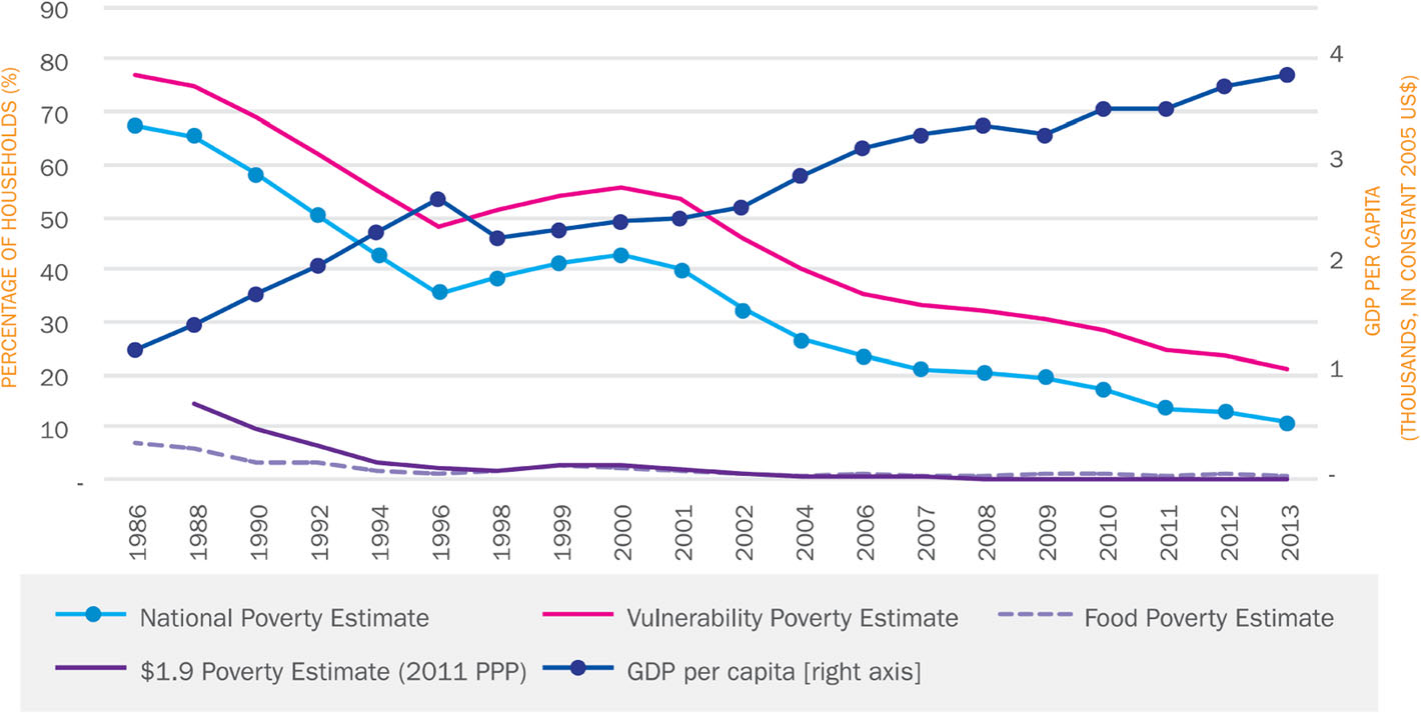
By all measures of poverty, Thailand has made impressive progress in poverty reduction [21]

Economic growth is the main driver for poverty reduction in Thailand. Per capita Gross Domestic Product (GDP) increased from US$ 1084 in 1986 to 3415 in 2013 (2005 constant US$). Extreme poverty as measured by the international extreme poverty line (US$ 1.90 per day, 2011 PPP) is no longer a concern, as it fell from 14.3% in 1988 to 0.1% in 2012. Using a national poverty line (in 2013, approximately US$ 6.20 per day 2011 PPP), the poverty head count also fell from 67% in 1986 to 10.5% in 2014, with 26.8 million Thai citizens moving out of poverty (Fig. 6) [21]. Evidence shows that since 2000, economic growth and improvement in income redistribution have played a dominant role in poverty reduction. It is found that nearly 85% of poverty reduction was attributable to economic growth while the remaining 15% was attributable to improvements in income redistribution. Further analysis between 2006 and 2013 shows that growth has been highly pro-poor, with redistribution playing a larger role, primarily through the introduction of elderly pensions and UHC [21]. This evidence explains why poverty levels prior to household out-of-pocket payments are low. Further, the low level of out-of-pocket payment for health by households, as a result of comprehensive benefits package and zero co-payment, explains the low level of additional poor due to medical payments. This helps explain the phenomena in Figs. 4, 5 and 6.

Several factors synergistically contributed to the financial protection of households against catastrophic health spending and impoverishment.

Firstly, all three public health insurance schemes provide full financial coverage to their members and cover the full cost of services to healthcare facilities; this does not allow any co-payment or balanced billing from service users. Full financial coverage for health services therefore reduced OOP in households. Also, general taxation, the sole source of financing for UCS and CSMBS, is the most progressive source of health financing as the rich pay a higher direct tax in monetary terms than the poor [1]. This is a redistribution tool between the rich and poor. Only public sources of financing, and not a reliance on external donors, can sustain UHC in the long term. Full financial coverage is reflected in the percentage of domestic general government health expenditure (GGHE-D) to current health expenditure which increased from 65% in 2002 (when the UCS was launched) to 78% in 2016; while the percentage of OOP on current health expenditure reduced from 28 to 12% during the same period [22]. The lower the proportion of OOP in financing health services, the lower the incidence of catastrophic health spending and impoverishment [23].

Secondly, the benefits package covered by all schemes is comprehensive, with no maximum limit of financial coverage and no co-payment at point of service, resulting in a massive reduction of OOP for households. The benefits package also applies a negative list approach; that is, all interventions are covered except a few exclusions such as infertility, aesthetic surgery and treatment under research or a pilot study [1]. In 2006, when the national capacity to conduct health technology assessments improved, more cost-effective interventions were included in the benefits package, which further boosted financial risk protection [16]. Curative services included medicines on the national list of essential medicines (NLEM) and in 2004 the NLEM was scaled up from the minimum ‘essential medicine list’ (with reference to the WHO model list) to a ‘reimbursement list’ for all three public health insurance schemes [16]. As of 2017, there are 849 drug items on the current NLEM [24], Table 4.

**Table 4:** Number of drugs in the national list of essential medicines, by 17 groups

Group no.	Category	No. of drugs
1	Gastrointestinal	39
2	Cardiovascular	72
3	Respiratory	30
4	Central nervous systems	102
5	Infections	133
6	Endocrine systems	43
7	Obstetrics, gynaecology and urinary-tract disorders	22
8	Malignant diseases and immuno-suppression	56
9	Nutrition and blood	93
10	Musculoskeletal and joint diseases	24
11	Eye	41
12	Ears, nose, oropharynx and oral cavity	42
13	Skin	47
14	Immunological products and vaccines	24
15	Anaesthesia	31
16	Antidotes	33
17	Contrast media and radiopharmaceuticals	17
Total		849

Source: Food and Drug Administration (FDA), Thailand [24]

**Fig. 7:**
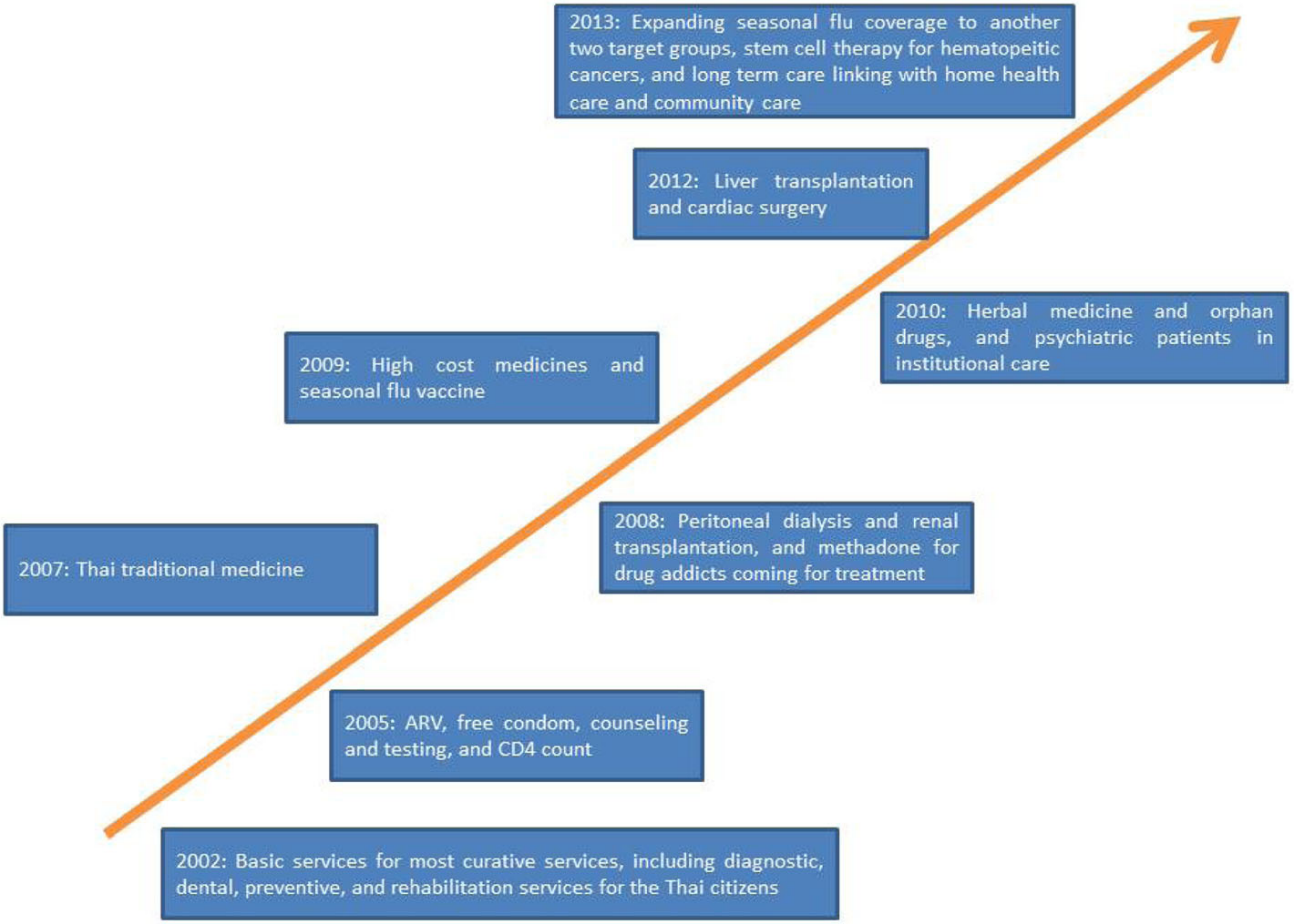
Historical evolution of the extension scope of the UCS benefits package

Thirdly, closed-end provider payment, notably the dominance of capitation for OP care and Diagnostic Related Groups under the global budget for IP care, is applied by the three schemes (except the fee for service for CSMBS OP services). This results in cost containment which frees up budget for the extension of the benefits package to further strengthen financial risk protection [1]. The UCS covers certain high-cost life-saving interventions such as antiretroviral treatment for HIV in 2006 and renal replacement therapy in 2009 (chronic dialysis is not cost effective, but the cost of dialysis is prohibitively high and can be catastrophic to households) [25, 26]. The UCS also covers long-term community interventions such as treatment for psychotic diseases, certain items in Thai traditional medicine, and seasonal influenza vaccinations [27]. Figure 7 describes the chronological events of the extension of the UCS benefits package to high-cost interventions, which were all subject to rigorous health technology assessment.

Fourthly, Thailand has developed local capacities to generate evidence on health technology assessments. Health technology assessments help improve the efficiency of resource use and minimize waste from spending on interventions that are not cost-effective. It was rigorously applied to the annual review for the inclusion of new health interventions into the UCS benefits package. The benchmark for including cost-effective interventions is the incremental cost-effectiveness ratio equal to one GDP per capita for one Quality Adjusted Life Year gained from the intervention. According to WHO’s recommendations, very cost–effective interventions should take less than an average per capita income for averting one disability-adjusted life year (DALY), and cost–effective interventions refer to the interventions that use resources less than three times average per capita income per DALY averted. In contrast, not cost–effective interventions are those with the cost exceeding three times average per capita income per DALY. Alongside value for money, which is measured by the cost effectiveness ratio, other criteria for decision-making are equally important. They include budget impact assessments, which should meet the criteria of being within the state’s fiscal capacity to fund new interventions and the readiness of health system to deliver the interventions equitably [28]. Assessment of these criteria ensures smooth implementation of new interventions.

Fifthly, capitation payment requires UCS members to register with a primary health care network, which comprises 1 district hospital and 10–12 sub-district health centres, serving about 50,000 people in the district catchment area [1, 3]. The gatekeeping function of a primary healthcare contractor network gains efficiency and provides better continuity of care for non-communicable diseases (NCDs) in particular. Better access to a primary healthcare network, with assured referral to provincial tertiary care hospitals when clinically indicated, results in adequate use of services and low level of OOP and transport cost by households [3, 26].

Lastly, the full geographical coverage of over 9800 sub-district health centres in all 8860 sub-districts, and 780 district hospitals and 116 provincial/regional hospitals in all 998 districts and 77 provinces is the solid platform for equitable access to the comprehensive benefits package which results in favourable financial risk protection at sub-national level [3, 4].

Certain limitations remain. Firstly, data on OOP paid by households is an aggregate figure which does not identify types of health facilities; this hampers further detailed breakdown analysis by types of health facility. Secondly, as the unit of analysis is ‘household’ not ‘individual’, per capita expenditure was estimated from total household OOP divided by the number of household members without adjustment; this cannot perfectly represent the real data collected from the individual household member. Lastly, the interview survey is prone to recall bias, which may undermine the accuracy of reported data by household members. Also, there was a possibility that the head of a household, who is the respondent to the NSO surveys, may not catch up with the real health spending by other household members.

## Conclusion

Thailand has been successful in reducing the incidence of catastrophic health spending and healthcare impoverishment. This success is ascribed to numerous factors, which provide good lessons for low- and middle-income countries for their quest towards the progressive realisation of UHC. The comprehensive benefits package, including high-cost interventions notably chemotherapy, radiation therapy, dialysis and anti-retroviral treatment, and no co-payment at point of care, contributes to the low level of OOP and low incidence of catastrophic spending and impoverishment. The benefits package also contributes to a high level of financial risk protection while strategic purchasing contributes to cost containment and health systems efficiency. Zero co-payment is possible as insurance funds provide full cost subsidies to healthcare providers. The full cost subsidy is achievable because of political commitment to health by increasing fiscal space for health, as reflected in a large proportion of domestic general government health expenditure to current health expenditure. The design of strategic purchasing by the insurance funds and the foundation of primary healthcare networks operated by an adequate number of qualified health workers nationwide are enabling factors for pro-poor healthcare utilisation and high level of financial risk protection reflected by the noteworthy reduction of catastrophic health spending, from 6% in 1996 to 2% in 2015, and impoverishment from the medical bills using national poverty line from 2.2% in 1996 to 0.3% in 2015. The full geographical coverage of health delivery systems, in particular primary healthcare at district and sub-district level with provincial hospital referral backups, is a solid platform for the equitable utilisation of health services by all.

## Data Availability

The data that support the findings of this study are available from the NSO but restrictions apply to the availability of these data, which were used under license for the current study, and so are not publicly available. Data are, however, available from the authors upon reasonable request and with permission of the NSO.

## Abbreviations

CSMBS: Civil Servant Medical Benefit Scheme
FDA: Food and Drug Administration
GDP: Gross Domestic Product
CHE: Current Health Expenditure
GGHE-D: Domestic general government health expenditure
NCDs: Non-communicable diseases
NELM: National list of essential medicines
NSO: National Statistical Office of Thailand
OOP: Out-of-pocket payment
PPP: Purchasing power parity
SDG: Sustainable Development Goals
SES: Socioeconomic surveys
SHI: Social Health Insurance
UCS: Universal Coverage Scheme
UHC: Universal Health Coverage
WHO: World Health Organization
